# Applications of Computer Vision on Automatic Potato Plant Disease Detection: A Systematic Literature Review

**DOI:** 10.1155/2022/7186687

**Published:** 2022-11-14

**Authors:** Natnael Tilahun Sinshaw, Beakal Gizachew Assefa, Sudhir Kumar Mohapatra, Asrat Mulatu Beyene

**Affiliations:** ^1^Department of Software Engineering, CoE for HPC and BDA, AASTU, Addis Ababa, Ethiopia; ^2^School of Information Technology and Engineering, AAiT, Addis Ababa, Ethiopia; ^3^Faculty of Emerging Technologies, Sri Sri University, Cuttack, Odisha, India; ^4^Department of Electrical and Computer Engineering, High Performance Computing and Big Data Analytics Center of Excellence, Addis Ababa Science and Technology University, Addis Ababa, Ethiopia

## Abstract

In most developing countries, the contribution of agriculture to gross domestic product is significant. Plant disease is one of the major factors that adversely affect crop yield. Traditional plant disease detection techniques are time-consuming, biased, and ineffective. Potato is among the top consumed plants in the world, in general, and in developing countries, in particular. However, potato is affected by different kinds of diseases which minimize their yield and quantity. The advancement in AI and machine learning has paved the way for new methods of tackling plant disease detection. This study presents a comprehensive systematic literature review on the major diseases that harm potato crops. In this effort, computer vision-based techniques are employed to identify potato diseases, and types of machine learning algorithms used are surveyed. In this review, 39 primary studies that have provided useful information about the research questions are chosen. Accordingly, the most common potato diseases are found to be late blight, early blight, and bacterial wilt. Furthermore, the review discovered that deep learning algorithms were more frequently used to detect crop diseases than classical machine learning algorithms. Finally, the review categorized the state-of-the-art algorithms and identifies open research problems in the area.

## 1. Introduction

Computer vision (CV) is a field that encompasses the use of various technologies such as AI, pattern recognition (PR), image processing (IP), and machine learning (ML) to provide object recognition, identification, and detection in a variety of application domains [1]. Though the definition varies depending on the problem and application domain, the commonly accepted definition of CV is “image analysis to extract data for controlling a process or activity” [2].

CV can be used in a variety of applications [3]. The following are the most common CV applications in agriculture: sorting and grading of fruits and vegetables [1, 4, 5], plant disease detection [6–9], plant disease classification [10–12], and quality inspection of fruits and vegetables [13–15].

Potato plant has been widely used and treated as one of the major crops in many developing countries to achieve food security strategies. However, the potato crop is affected by several diseases. As a result, this paper investigates the application of CV in potato disease detection by using a systematic literature review (SLR). In addition, the review analyzes the major diseases that affect potatoes and the various CV-based detection techniques.

The need for this SLR arose from the demand for categorizing relevant existing works related to the area of research using appropriate methodology. Consequently, other researchers will be able to comprehend the current works, as well as future trends in the field [16].

More specifically, in this review, therefore, three sets of review questions are formulated to uncover what major potato diseases exist, how these diseases are detected using CV-based techniques, and which CV-based algorithms are used in potato disease detection. The the review questions developed in this SLR to get the state-of-the-art are as follows:

  RQ1: Which plant diseases affect potato crops?
  RQ2: How do potato plant diseases are detected using CV-based techniques?
  RQ3: Which CV-based algorithms are the most widely used in potato disease detection?

In this review, the papers published from 2016 to 2022 are considered.

Ultimately, the main contributions of this work are as follows:

- Identification of the major plant diseases that affect potato plants
- A thorough explanation of CV and how it can be used to detect potato diseases
- A list of CV algorithms that are commonly used in potato disease detection
- Typical open research areas in the application of CV for potato disease detection

The remaining sections of this paper are organized as follows.  Section 2 explains the methods employed to conduct this SLR.  Section 2.1 describes the steps followed in conducting the review and the inclusion and exclusion criteria applied to select the primary studies (PSs).  Section 2.2 describes review protocol formulation, PSs source selection, and search procedure, and finally, data extraction and quality assessment. Subsequently, Section 3 describes the four major techniques and algorithms used for potato disease detection. Accordingly, Section 3.5 explains about the deep CNN algorithm, Section 3.6 discusses the major pretrained models used for potato disease detection, Section 3.7 describes the most widely used ML algorithms in potato disease detection, and Section 3.8 explains about the graph cut segmentation technique.  Section 4 pinpoints related works done in the area of potato disease detection. Following that, Section 5 gives a comprehensive overview of open research challenges and future trends. Finally, Section 6 summarizes the review.

## 2. Methodology

### 2.1. Steps of Conducting the SLR

**Figure 1:**
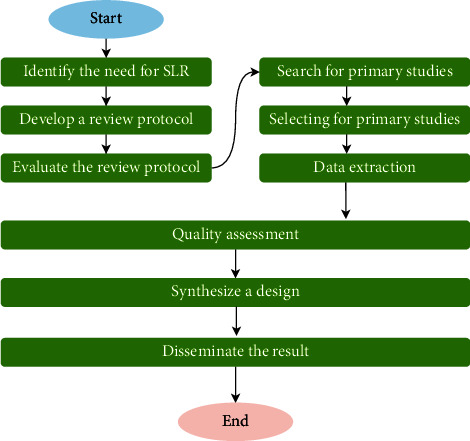
SLR steps.

The primary activities carried out during this SLR are classified into three steps: (i) planning the review, (ii) conducting the review, and (iii) writing the review report. In fact, before planning the SLR, one must first address the specific inclusion and exclusion criteria of the various previously done studies. In Figure 1, the steps used to produce the SLR are depicted.

In SLR, the primary studies must meet inclusion criteria to be chosen as relevant paper that answers a review question. In order to do so, the criteria for inclusion and exclusion must be applied. The inclusion criteria are a set of requirements a paper must meet to be selected as a relevant paper, whereas a paper that does not fulfill the requirements is excluded based on the exclusion criteria. The proposed criterion is adopted based on the guideline [16] and depicted in Table 1.

### 2.2. Formulation and Validation of the Review Protocol

By having a predefined review protocol that specifies the steps of conducting an SLR, there is less room for bias and unfair selection of primary studies. In other words, failure to construct a review protocol could possibly bring an impact on the review since a researcher may choose studies depending on his/her expectations [16]. Some of the most important components of a review protocol are as follows, among others:

- The review question
- Background or the rationale of the survey
- The search strategy in which the PSs are going to be selected
- Study selection criteria which consist of inclusion and exclusion criteria
- The study checklist and procedures that assure how much helpful the selected studies are

#### 2.2.1. Source Selection and Search Procedure

Conducting an SLR requires having a searching mechanism that achieves the best result. To do so, keywords have a great impact on the search and retrieval of primary studies. Additionally, selecting relevant keywords gives the ability to filter out unwanted papers. It also shows gives relevant papers a higher ranking. The search strings formulated for finding relevant primary studies are mentioned in the following:

  (Potato Disease) AND (Identification OR Detection) AND (Computer Vision)
  AND (Technique OR Methods OR Algorithms)

#### 2.2.2. Primary Study Selection (PSS) Process

All papers found during the PS searching process cannot be considered equally relevant. Hence, a method is needed to select those relevant papers. In this review, the PSs are selected using the following six steps.

Step 1. Selecting a digital library based on the domain of study.
Step 2. Define a search string that can be used to search papers from the selected digital libraries.
Step 3. Execute a pilot search for retrieving papers. If the majority of known papers are found, then go to Step 5 which retrieves the initial PSs; if not, go to Step 4 which is refining a search string.
Step 6. Exclude all primary studies based on the title, abstract, and full text. Finally, all relevant papers are retrieved.

**Figure 2:**
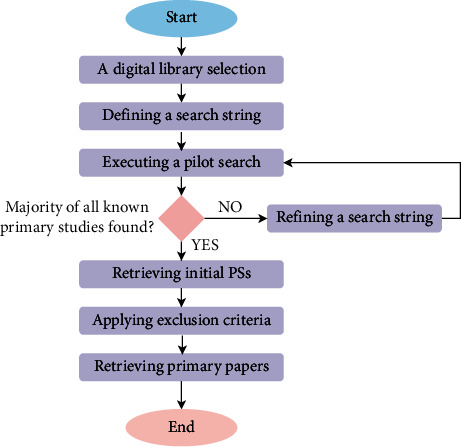
PS selection steps.

The primary study selection procedure is extremely vital in order to maintain the quality of the SLR. This is accomplished by outlining which primary studies should be selected, how the search string should be employed, and what standards to be used for the inclusion and exclusion of primary studies. Therefore, in order to make a review less biased, it is important to document all the processes and follow the SLR steps.  Figure 2 shows the primary study selection procedure.

To find PSs, the process begins by selecting a search engine and searching for papers using the formulated search string. Subsequently, apply the exclusion criteria to the result that follows. The initial paper selection process retrieved 80 results, but after the inclusion criteria were applied, only 52 papers were left. The snowball and manual search methods were used to find additional papers. Finally, the procedure ends with the selection of 39 primary studies based on the updated inclusion and exclusion criteria as well as a quality assessment (QA) checklist. The summary of the process of selecting the 39 primary studies is depicted in Table 2.

The review questions listed in Section 1 had to be addressed based on the information found in the 39 primary studies selected.  Table 3 presents the primary studies that were chosen for this study along with a breakdown of those papers which addressed RQ.

**Figure 3:**
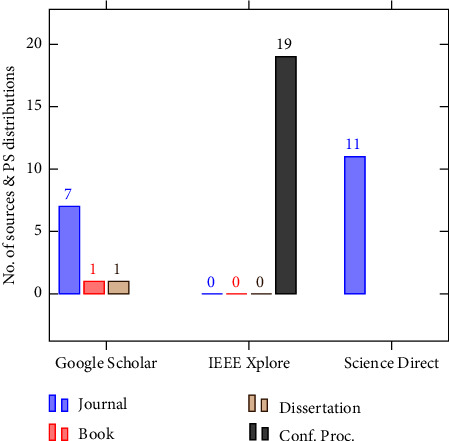
Sources of PSs and their distributions.

This review included primary studies from reputable journals and conference proceedings. This ensures the quality of the review by weeding out any subpar or unreliable sources.  Figure 3 demonstrates the relationship between the source of the primary studies and their distributions. As the figure depicts, the majority of the primary studies came from conference articles (19) and journals (18), with a smaller number coming from doctoral dissertations (1) and books (1).

The number of primary studies found before and after the PS selection process is shown in Table 4. These studies were found in three publication sources (Google Scholar, IEEE Xplore, and ScienceDirect).

#### 2.2.3. Data Extraction and Quality Assessment

The quality of the data acquired from the study is significantly improved through data extraction and a quality assessment (QA) checklist. Moreover, a data extraction form has developed and been used to retrieve data from the PSs. As most review papers recommend, depending on the type of study, a checklist should be formulated because it helps to extract relevant and accurate information from primary studies. The data extraction form prepared is presented in Tables 5 and 6.

There are numerous methods for assessing the quality of primary studies, one of which is a binary scale. It is used in this study because the review is not interested in providing rating for studies but in identifying whether an article's evaluation is positive (yes) or negative (no) for a given QA question. The QA questions are mentioned as follows based on Ref. [16]: (A) Is there a clear description of the aim of the study? (B) Are the aim and purpose of the study addressed through evaluation? (C) Is the target selection of documents/cases well defined? and (D) Is the evidence presented sufficient to support the claim?.  Table 7 presents the QA questions.

## 3. Major Findings

The goal of this review is to answer the review questions (RQs) posed in the preceding section using the PSs that were identified. The major findings extracted from the 39 PSs are presented below.

### 3.1. RQ 1: What Major Diseases Affect Potato Crops?

The economy of developing countries is heavily reliant on agriculture where the majority of the population works as farmers. Therefore, the agriculture sector plays a major role in the country's food security and GDP [55]. One of the major causes of the reduced quality and quantity of agricultural products is plant disease. Pathologists classify plant disease by parts of the plant like the root, kernel, stem, and leaf. However, most symptoms that appear on the leaf parts are responsible for the reduction of crop production in quality and quantity. On the other hand, plant diseases are classified into two groups based on their causes: parasitic and nonparasitic. Pathogens, pests, and weeds are all parasitic causes of plant disease, whereas nonparasitic causes include water, temperature, irrigation, and nutrients [18].

Even though it has a promise for food security programs in most developing countries, there is a low yield of potato production. There are many factors for this reduction where the main ones are diseases like late blight [20] and insects like tube moths. According to research, the estimated loss of potato crops due to late blight ranges from 6.4% to 61.7% depending on crop variety [21]. Furthermore, potatoes can be affected by diseases caused by viruses, like early blight, and other microorganisms. Potato disease has a significant impact on growth and crop yield. The impact spans social, ecological, and economic dimensions. Therefore, early detection and treatment of plant diseases are important to the growth and yield of many agricultural products. Here, the major potato diseases and their symptoms are identified and discussed.

Bacteria wilt is caused by the bacteria *Ralstonia solancearum*. It is capable of infecting not just potatoes but also other plants such as chili, tomato, tobacco, and eggplant, as well as various weed species. In some areas, the disease is the major cause of reduced productivity, and it is particularly damaging in places like Shashemene, Ethiopia [17]. Symptoms of an infection on a plant can vary. They usually start at the tips of the leaves or where the stems branch out and then spread to the rest of the plant. When the leaves become yellow at the base, the plant wilts and die. A blue cooling ring emerges when the stems are sliced. Mildly infected tubers will not show any visual signs of the disease since symptoms are hidden from view [17].

Late blight, caused by the fungus named *Phytophthora infestans*, is a major global threat to potatoes and related crops [18]. It starts infecting plants from the tuber initiation stage till harvest, causing crop failure on a regular basis. The rain, humidity, and cold temperatures can make infections more common and challenging to treat. The symptoms of this disease are different in different parts of the plant. The different parts of the plant, such as leaves, stems, and tubers, are affected by this disease [56]. The disease has the ability to spread swiftly, and if proper precautions were not taken, the plant might perish in two or three days, perhaps destroying the entire field, depending on the crop variety [20]. In the case of the white powder on the damaged leaves, the disease can be transmitted by wind and infect other plants [17]. The summary of RQ1 findings and analysis are presented in Table 8.

### 3.2. RQ2: How to Detect Plant Disease Using CV?

Due to the significant overlap in the techniques used in CV and image processing, many scholars in the field use these terms interchangeably [57, 58]. CV is a combination of image processing and pattern recognition [59, 60] where the final output is image understanding [61].

Images carry a vast amount of information consisting of finite elements where each has a particular location and value. These elements are called picture elements or pixels. We, humans, are very selective about what is consumed based on the visual senses. One of the tools used to extract information from images is digital image processing, which manipulates them electronically. Formally, it is defined as “a method of enhancing and extracting valuable information from a digital image using digital computers.” It involves converting an image to a digital form and uses various operations to enhance the image including smoothening, sharpening, and color correcting.

An image is simply a representation of an object, person, or scene. To define simply, a digital image is a two-dimensional function *f* (*x*, *y*) that is a projection of a three-dimensional scene into a two-dimensional projection plane, where *x* and *y* represent the location of the pixel which has an intensity value. Pixel coordinates may be represented using vector notation. By convention, each vector is vertically oriented while its transpose is horizontally oriented [24]:

(1) $$\begin{array}{c} X=\left[\begin{array}{c} X\\ Y \end{array}\right]={\left[\begin{array}{cc} X & Y \end{array}\right]}^{T}=\left(X,Y\right)\text{.} \end{array}$$

Equation (1) is the pixel representation using vector notation.

Mathematically, an image is a matrix representation of a 2D image using a finite number of pixels. Each pixel has a numerical value representing three types of images: gray-scale, color, and binary.

### 3.3. Levels of Digital Image Processing

Digital image processing can be used to extract information from a digital image and analyze it. To fully exploit digital image processing, it is divided into three levels: low-, mid-, and high-level processes.

Low-level processing involves primitive operations such as image preprocessing to remove and reduce noise, contrast enhancement, image sharpening, and image resizing, among others. The main major goal of this primitive operation is to improve the nature of the image to get better information. Both the input and the output of low-level processing are images.

Mid-level processing contains activities such as image segmentation, image description, and object recognition. Here, the input is a processed image, and the final output is a feature or attribute of, for example, edges, contours, and regions, extracted from the image [24].

High-level processing involves complex image processing tasks to “make sense of” the collection of identified objects. The tasks of this level vary depending on the nature of the CV problem at hand. In this level of processing, the input is a set of attributes, and the output is an understanding of the digital image based on the extracted information [24, 62].

Image processing has a broad array of applications. Here are some examples. Medicine where the inspection and interpretation of medical images obtained from, like, a CT (computed tomography) scan, positron emission tomography (PET), and MRI (magnetic resonance imaging) are done. Agriculture is another potential application area of image processing where, for example, capturing satellite/aerial views of the land is conducted to detect and classify plant diseases. Furthermore, it can be used to determine how large a given area is for various purposes. Various industries use image processing to automate different tasks, including advanced quality control of products, reducing safety risks, and increasing productivity. Another application area is law enforcement. Here, image processing can be used in crime prevention and investigation by employing various biometrics techniques like fingerprint identification, facial recognition, and iris detection [23].

Tadmare and Mahalakshmi [28] explained the causes of plant disease into two general categories as living agents and nonliving agents. In the living agent category, the causes of plant disease are bacteria, fungi, and viruses. In the nonliving agent category, the causes are temperature, humidity, soil type, and others. The authors also mentioned the processes and steps required for plant disease detection.

One of the application areas of CV is plant disease detection where specific plant diseases are identified based on information gathered from leaves, stems, and roots of the plants. Typically, two phases are required to build an automatic plant disease detection system. The first phase is called segmentation, which is the process of dividing an image into segments. These segments are then used to detect disease-infected parts. The second phase is called feature extraction which divides and reduces the initial set of raw data into more manageable groups. This phase helps to get prominent features from large raw data. Following this, the classification is applied choosing from the classifiers available [23, 24].

Sharif et al. [26], Prakash et al. [27], and Singh and Misra [63] explained the basic steps to build a model or create a system using image processing. The general steps required for building an image processing system are image acquisition, image preprocessing, image segmentation, feature extraction, feature selection, classification, and performance evaluation.

The steps many authors followed start from an image dataset preparation. This step is known as image acquisition. Following that, the next step is image preprocessing, which involves performing primitive operations on the acquired image such as cropping, resizing, and other processes. Subsequently, image segmentation is used to divide a digital image into many segments (pixels). This makes it easier for image classifiers to analyze the image further. The feature extraction phase helps to identify features that express a given image in a meaningful way. Then, feature selection reduces a large set of features by selecting only the effective ones. Finally, image classification or detection is performed to detect and classify images into different classes.

An image processing system takes a large number of images dataset to detect and classify plant diseases. These images can be taken from benchmarked datasets or by capturing the images using a high-quality camera [28]. After an image is taken, it goes through a series of steps, depending on the methods and techniques employed in the study. Finally, the system's performance is evaluated using evaluation metrics.

Most farmers use naked-eye observation to detect plant diseases. This is not efficient in identifying the exact pathogen because it is highly dependent on the knowledge and experience of farmers. Moreover, in a large agricultural field, it would be too difficult to identify the diseases affecting the crops. Advanced technology, such as machine learning or deep learning, can be used to solve these problems because the technology is capable of automatically detecting and classifying plant diseases with better speed, accuracy, and affordability [28, 64]. Generally, developing an early plant disease detection and diagnosis system would help farmers minimize huge losses in crop production. The summary of RQ2 is presented in Table 9.

### 3.4. RQ3: Which CV-Based Algorithms Are Most Widely Used in Potato Disease Detection?

There have been many studies on the use of CV methods or algorithms to identify and classify plant diseases.

In this major section, CNNs, transfer learning, ML algorithms, and graph cut segmentation are discussed.

### 3.5. CNNs (Convolutional Neural Networks a.k.a ConvNets)

Deep learning is defined as the use of artificial neural networks that contain successive layers as opposed to traditional neural network methodology. The term “deep” indicates that in deep learning, there are more layers than machine learning techniques [53]. This is revolutionary. Besides its computational feasibility, it gives much better results in areas like image recognition, voice recognition, and other complex operations involving quite large data. Each output layer in deep learning is used as an input for the next layer.

Even though deep learning began a few years back, it already achieved huge success compared to some other areas of study. The field is being used in many application domains. Generally, the learning process of deep learning can be unsupervised, supervised, or semisupervised based on the nature of the problem at hand.

Deep CNN has shown interesting performance results in CV and machine learning problems [65]. The use of multiple feature extraction stages that can automatically learn a representation from a given input data is the key reason for using deep CNNs. Furthermore, the characteristic of deep learning is that it “does not divide the feature extraction and classification” as separate tasks. That is because deep learning models automatically learn features while training [66]. Factors like the availability of a massive amount of data and the constant improvement of hardware technologies have contributed to the advancement of CNN research. In recent years, several interesting CNN architectures were reported. These architectures use different loss functions, activation functions (AFs), parameter optimization, and architectural innovation, among others [67].

Lu et al. [68], Reddy et al. [69], and Amara et al. [70] used a deep learning algorithm called CNN. It constitutes powerful techniques for modeling complex processes to perform pattern recognition applications using a large amount of data [71]. Additionally, Mehdipour Ghazi et al. [40] explained how CNN is different from other handcrafted feature extraction methods like texture analysis, followed by random forest and support vector machine. The difference between the above-mentioned approaches and CNN includes those as follows: (i) CNNs do not require expert-based feature extraction, (ii) CNN architectures do not require segmentation of features by human experts, and (iii) CNNs need lots of data since it has millions of learnable parameters. Nonetheless, this problem can be solved by “data augmentation” or by using a “pretrained model.”

CNNs are widely used neural networks that solve problems related to image identification, object recognition, image classification, face recognition, and others. Furthermore, it can detect and classify objects with minimal preprocessing achieving a higher result when analyzing objects. Moreover, it is simpler to separate features in multilayered objects [67].

CNN learning architectures are highly dependent on the data provided by the algorithm, which is finally used for applications like forecasting or classification. The algorithm computes future maps through the use of AFs [46]. Mathematically, the function is defined as

(2) $$\begin{array}{c} y_{j}^{l}=f\left(z_{j}^{l}\right)\text{,} \end{array}$$

where *y*_*j*_^*l*^ is called the future map and *f*(*z*_*j*_^*l*^) is called the AF.

CNNs store a given dataset using a 2-dimensional convolution operation. The length of the output (*O*) is mathematically calculated as [72]

(3) $$\begin{array}{c} O=\frac{\left(W-F+2P\right)}{\left(S+1\right)}\text{,} \end{array}$$

where *W* stands for input length, *F* for filter size, *S* for stride, and *P* for padding.

**Figure 4:**
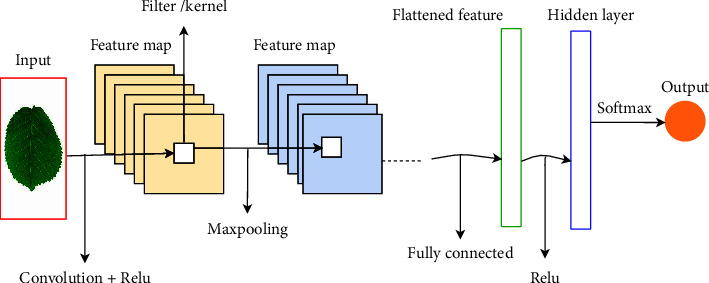
Sample CNN architecture.

Generally, as Figure 4 depicts, a typical neural network has four main layers: convolutional layer, pooling layer, AF, and fully connected layer [73].

#### 3.5.1. Convolutional Layer

**Figure 5:**
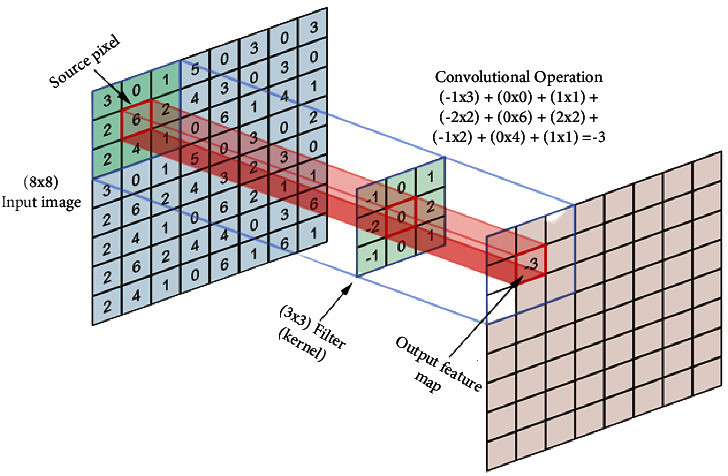
Convolution operation.

CNN took its name from the convolution layer. In this layer, the matrix operation is performed to extract feature maps from the input image [69]. This mathematical operation is depicted in Figure 5. First, the filter is shifted step by step starting from the upper left corner of the image. At each step, the values in the image are multiplied by the values of the filter (kernel), and the result is summed up. A new matrix with a smaller size is created from the input image.

Mathematically, a convolution is defined as a product of functions *f* and *g* that are the objects in the algebra of Schwartz functions in Rn [74].A convolution of two functions *f* and *g* over infinite range [−*∞*, *∞*] is given by

(4) $$\begin{array}{c} \left(f*g\right)\left(t\right)\overset{\text{def}}{=}\int_{-\infty }^{\infty }f\left(\tau \right)g\left(t-\tau \right)d\tau \text{,} \end{array}$$

where (*f∗g*) (*t*) denotes the convolution of functions *f* and *g*.

#### 3.5.2. Pooling Layer

**Figure 6:**
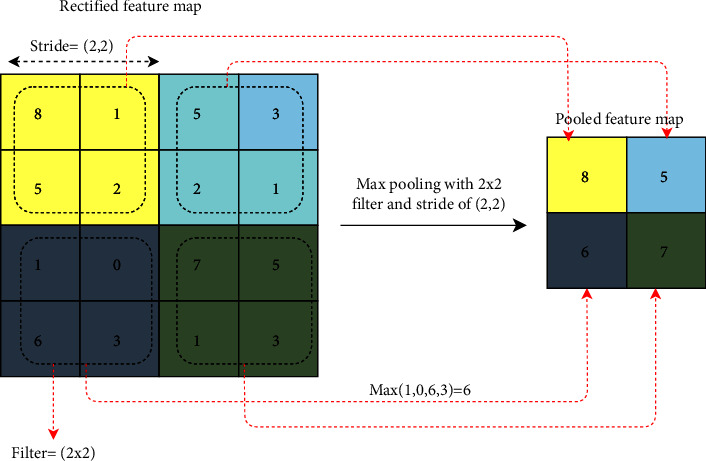
Max pooling.

The pooling layer operation begins after the convolutional layer operation is completed. Pooling, also known as downsampling, is a fascinating operation. It takes similar information from the local neighborhood's receptive fields and generates the dominant response within this local region [69, 73]. To do so, the operation employs the following functions: max pooling, average pooling, and sum pooling, to name a few. The max pooling, for example, performs operations by selecting the largest element from the input matrix concerning the filter. The operation is illustrated in Figure 6.

#### 3.5.3. Activation Function (AF)

An AF is used as a decision function helping to learn complex patterns. It affects the convergence speed of neural networks. Since there are many types of AFs, selecting the right one deserves a critical decision as this will affect the performance of the neural network [73].

Sardogan et al. [29], Reddy et al. [69], Yadhav et al. [76], and Afework and Debelee [77] used different AFs such as sigmoid, tanh, maxout, SWISH, ReLU, and variants of ReLU such as leaky ReLU, ELU, and PReLU to inculcate a nonlinear combination of features. The main reason to use AFs is that without them a neural network would become a polynomial function with degree one which is a linear regression equation. However, among the mentioned AFs, ReLU and its variants are preferred because they solve the problem of vanishing gradient. Among the recently proposed AFs, MISH shows better performance when compared with ReLU in most of the currently used benchmark datasets [78]. Among the mentioned AFs, Softmax, Relu, and Sigmoid are selected, based on the popularity of use and are explained as follows.

*(1) Softmax AF.* It is mostly used in the output layer of deep learning algorithms to make decisions based on the input variable's weight. Mathematically, Softmax AF is defined as

(5) $$\begin{array}{c} \sigma {\left(\overset{⟶}{z}\right)}_{i}=\frac{e^{z_{i}}}{\sum_{j=1}^{K}e^{z_{j}}}\text{.} \end{array}$$

*(2) ReLU (Rectified Linear Unit) AF*. The ReLU AF and its variants are the most widely used in many deep learning studies [79]. The ReLU AF is represented as

(6) $$\begin{array}{c} f\left(x\right)=\max\;\left(0,x\right)=\left\{\begin{array}{cc} x & \text{if}x\geq 0\text{,}\\ 0 & \text{if}x<0\text{.} \end{array}\right. \end{array}$$

*(3) Sigmoid AF.* It is one of the most common AF [79] which uses a probabilistic approach to make decisions with values ranging from 0 and 1. Mathematically, Sigmoid AF is defined as

(7) $$\begin{array}{c} G\left(z\right)=\frac{1}{\left(1+e^{-z}\right)}\text{.} \end{array}$$

#### 3.5.4. Fully Connected (FC) Layer

**Figure 7:**
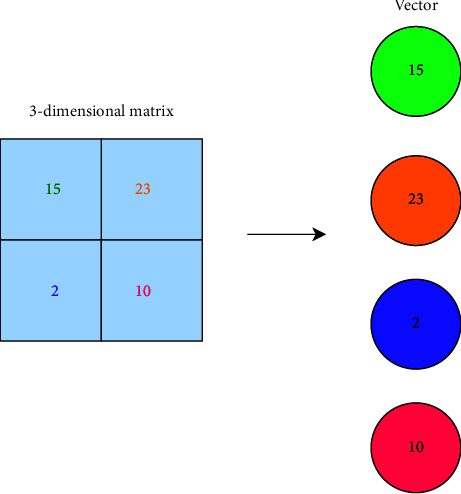
FC layer operation.

The FC layer is a simple feed-forward neural network. The output of the final pooling or convolutional layer, which is flattened, becomes an input to the FC layer. The term “flattening” is used to describe the process of converting the three-dimensional matrix output of a pooling or convolutional layer into a one-dimensional array.  Figure 7 demonstrates the process of converting a 3D matrix to a 1D array.

### 3.6. Transfer Learning

One of the best-known strengths of deep learning is its ability to perform better at solving complex problems, which is why it is so widely recognized and used. Transfer learning is one of the methods which has made the field more powerful. Simply, it is reusing knowledge gained from training data and applying it to a different but related problem [80, 81]. The method is commonly used when there is a new dataset that is smaller than the original dataset that was used to train the pretrained model [65]. In most cases, the approach improves the model's performance. Some of the widely used transfer learning techniques via pretrained models are explained below.

AlexNet is a deep CNN model trained on 1.2 million images under 1000 classes from the ImageNet Large Scale Visual Recognition Challenge (ILSVRC) dataset. It was the winner of the competition in 2012. The architecture has about 650,000 neurons and 60 million parameters. Moreover, the components of the model are arranged in five convolutions, two normalizations, three max-poolings, three fully connected layers, and Softmax at the output layer. Dropout regularization was applied to minimize the overfitting problem, where in each convolution layer, the ReLU AF was used [39].

In summary, AlexNet uses ReLU nonlinear AF with a dropout of 0.5, stochastic gradient descent (SGD) with a momentum of 0.9, the initial learning rate of 0.01, and a reduction of 10 when validation accuracy became flat. This network employs L2 regularization with a weight decay of 0.0005 [82].

VGG19 is another type of pretrained deep CNN model developed by Simonyan and Zisserman [83]. The model trained for the ILSVR competition has more than 15 million tagged high-resolution images. To build the model, the dataset was partitioned into 1.3 million images for training, 50,000 images for validation, and 100,000 images for testing [39]. The largest VGGnet model has 144 million parameters from 16 convolution layers with a kernel size of 3 × 3, five max pooling with a size of 2 × 2, three fully connected layers, Softmax AF in the output layer, output regularization in the fully connected layers, and ReLU AF in the convolution layer [40].

GoogleNet is a pretrained model that won the 2014 ILSVRC competition. The objective of the GoogleNet architecture is to reduce computational cost [84]. The design of GoogleNet has increased the width and depth of the network while decreasing the computational cost.

Even though hyperparameter selection methods used in different works are not within the scope of this work, the method is one of the crucial tasks involved in developing machine learning-based models [85]. A hyperparameter is a machine learning parameter that is used to control how a model learns. It has different domains, for instance, a learning rate has a real value, the number of layers has an integer value, whether to use an early stopping or not has a binary value, and the choice of optimizer has a categorical value. For integers and real-valued hyperparameters, the domains are mostly bounded for practical reasons, with only a few exceptions [86–88].  Table 10 depicts the summary of some of the most widely employed pretrained models, and Table 11 analyzes the total amount of parameters used in potato disease detection.

### 3.7. Machine Learning Algorithms

Machine learning is a technique that enables a system to learn by itself from examples depending on which it can be used for decision-making. Machine learning algorithms are classified into three: supervised, reinforcement, and unsupervised. In different studies [38, 48, 49, 54], based on the problem nature, various machine learning algorithms are used for plant disease identification. Some of the most frequently used machine learning algorithms are support vector machine (SVM), random forest (RF), linear discriminant analysis (LDA), logistic regression (LR), decision trees (DT), k-nearest neighbors (KNN), and naive Bayes (NB). A summary of machine learning algorithms used in potato disease detection is presented in Table 12.

### 3.8. Graph Cut Segmentation

Image segmentation is the process of identifying and separating different objects in a given image based on some criteria [93, 94]. It is considered as one of the preprocessing activities in the field of object tracking, pattern recognition, CV, and other fields [95]. The goal of image segmentation is to simplify or change a given image as accurately as possible using as few steps as possible [96].

Graph cut is a very popular approach in a wide variety of CV-related problems. “It minimizes an energy function consisting of a data term, which is computed using color likelihoods of foreground and background, and a spatial coherency term.” A major drawback of the approach for image segmentation tasks is that it does not produce very accurate segmentation of thin elongated objects due to “shrinking bias” [96].

A comparative analysis of the algorithms used for potato disease detection is presented in Table 13.

**Figure 8:**
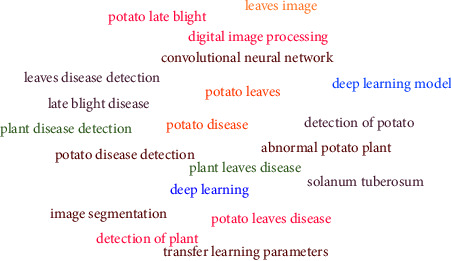
Frequency of word distribution.

To analyze the distribution of terms, a word cloud generator was used. The tool assesses the terms that are used most frequently. According to the result, in the selected primary studies, the most frequently appeared word is deep learning and the most frequently mentioned algorithm is CNN.  Figure 8 depicts the distributions of terms. Moreover, Table 14 presents the count and relevance of terms.

## 4. Related Works

Plant diseases reveal visual signs that assist in their identification and classification. This is used as one input for CV utilizing deep CNN algorithms.

Potato is among the most commonly consumed foods being ranked fourth worldwide [21]. Different pathogens cause plant diseases minimizing crop production. Generally, inadequate classification and late detection have harmed plant productivity. This section includes related works conducted on plants and, more specifically, on potato disease detection or classification.

Hirani et al. [98] experimented with a deep CNN algorithm to build a plant disease detection model. The experiment was based on an open-source dataset named PlantVilage that contains 87.9k images. The repository contains 38 types of plant disease pairs. The authors used 80% of the data used for training (70295 images) and 20% for validation (17572 images).

Barman et al. [99] used a Self-Built Convolutional Neural Network (SBCNN) and MobileNet model for potato leaf disease detection using a dataset that contains 2152 images. Additionally, the PlantVillage open-source dataset was used. The overall dataset contains three classes of potato diseases. The first two classes have 1000 images, and the third class has 152 images. The data augmentation technique was used with the third class to increase the total image to 1030. In addition, the authors claimed that both the SBCNN and MobileNet models performed well. Moreover, the model has been deployed to detect potato leaf disease using smartphones. Finally, the authors suggested two key points. First, using data augmentation methods to improve the performance of the models, and second, cautious while working with an imbalanced dataset which could lead to overfitting.

Singh et al. [100] mentioned the use of a homogeneous dataset that might cause challenges during testing. That is because the real cultivation area has a heterogeneous and complicated background. One of the constraints of using a public benchmark dataset for testing is that the model's efficiency usually suffers when tested in a real-world image. However, the authors in Ref. [98] used a public benchmark dataset with multiple deep learning methods to train the proposed model, including custom CNN, Inception-v3, spatial transformer network (STN), and large transformer network (LTN). They have shown that the transformer model outperforms the other models by 97.98%.

Microorganisms, genetic abnormalities, and disease agents like fungi, bacteria, and viruses are the cause of many plant diseases. Among the mentioned agents, the main reasons for the spread of potato disease are fungus and bacteria. As a result, the detection of these diseases is required. Hence, a plethora of authors developed an automatic potato disease detection system based on them [101].

Many previous researchers have proposed CV and image processing techniques to detect and classify plant diseases using local binary pattern (LBP) [102] and *K*-means [97]. Both works used a deep learning model to map functions and generate features. The amount of data they have used is 2152 leaf images from the PlantVillage open dataset repository. The dataset was prepared as three classes each of which contains images of early blight, late blight, and healthy potato leaves. The first two classes each contain 1000 images, and the third class contains 152 images. Furthermore, the dataset partitioning method used is 70–30 which means 70% of data (1700 images) is used for training and 30% (452 images) is used for testing.

Currently, plant disease detection using several image detection approaches is a huge research area in the field of agriculture. One of the driving factors of these researches is the visibility of diseases in the different parts of plants. These in turn drive the productivity of agricultural goods. Some of the tools used for achieving plant disease detection tasks are artificial intelligence, image processing, and CV. To be more specific, some of the algorithms used by previous authors are k-NN, CNN, SVM, and decision trees. Nonetheless, the potential advantage of CNN and R-CNN is not fully discovered [92].

In the works of [103], faster R-CNN and GoogLeNet algorithms were merged to detect pepper and potato leaf diseases. Furthermore, some photos were made up of two sections, and image stitching was used to merge them. This technique simplified image processing on those leaf images taken from a wider angle. To sum up, the authors targeted using GoogLeNet for improving the performance of Faster R-CNN [92].

Sert [92] used the PlantVillage image dataset for training together with other remaining leaf images collected from fields of pepper and potato. In general, a total of 544 images were used from the open repository and locally captured images. Moreover, in order to generate additional images, four data augmentation parameters were used increasing the total number of images to 2176. Subsequently, many algorithms have experimented including Fast R-CNN with AlexNet, GoogleNet, and SqueezNet. Accordingly, the Fast R-CNN with GoogleNet classifier scored the highest accuracy.

The impact of remote sensing with deep learning on crop growth and disease detection has shown improvements in agricultural production. The researchers in Ref. [104] have made an attempt to develop a technique to automatically analyze aerial images of potato crops using a deep neural network. Based on this, the researchers then developed a method that can automatically recognize healthy and stressed crops at the plant level.

Precision agriculture (PA) is currently one of the hottest areas of research with many countries attempting to adopt the technology to improve their agricultural production. The primary goal of this technology is to increase crop yield while reducing environmental impacts. Despite the obvious benefits of PA to agriculture production, and the economy, only a few countries have adopted the technology [105]. Some of the challenges to adopting the PA technology are the lack of advanced data processing methods and a platform for automated seeding, weeding, and harvesting as per the works of [104].

## 5. Open Research Challenges and Future Trends

Based on this SLR, the following open research challenges and future trends for further exploration are identified:

1. Even though there have been various studies on the use of CV in agriculture, notably for potato disease diagnosis, there are a handful of mobile-based applications that farmers can utilize. As a result, the models developed by various researchers need to be integrated with mobile platforms for further seamless usage and application by farmers.
2. The majority of researchers employed laboratory-prepared datasets that were collected with excellent brightness, contrast, locations, and other features. In practice, certain conditions may not be met throughout the dataset preparation. As a result, models trained using laboratory-prepared datasets may not result in a good performance in a real-world setting.
3. One of the major challenges when building a machine learning or deep learning-based model is setting the optimal value of hyperparameters. In many works, the hyperparameter selection methods or techniques are not explicitly discussed. To summarize, selecting an appropriate hyperparameter tuning method is critical to building a robust model with little experimentation and resources.
4. Analyzing the severity of diseases is helpful to make decisions. However, there are only a few researchers who have worked on how much the disease affected potato crops.
5. Performing a comparative analysis to assess the complexity of model implementation would benefit researchers when determining which model to use.

Previously, most researchers [28, 44, 49, 54, 62] employed machine learning methods to detect potato diseases. However, this review revealed that computer vision via deep learning algorithms is the most common approach. In particular, the CNN transfer learning technique has been used in a number of studies.

## 6. Conclusions

Potato is among the most consumed crops throughout the world, especially in developing countries. Furthermore, its contribution to achieving food security programs is considerably high. However, several diseases have affected the quality and quantity of its production. Due to this, many scholars have studied automatic potato disease detection algorithms using different CV and ML techniques. The results achieved were not only promising but also improving the quality and quantity of potato crop production. Despite the various research works and outcomes, it is difficult to know what has been done and what results were obtained. Moreover, it has become difficult to frame new research efforts capitalizing on existing works. Therefore, the contribution of this review is multifold. First, it attempted to identify which diseases affect potato crops. Second, it has analyzed the state-of-art methods and algorithms used to build potato disease detection models. Third, the review analyzed how CV is used in potato disease detection. Fourth, the review analyzed the state-of-the-art algorithms used to detect potato disease and which disease minimizes the total yield of crops. Finally, the review pinpointed the main open research challenges and future trends. The three key findings of the review are summarized as vis-a-vis the initial objectives and RQs.

Potato crops are affected either by biotic or abiotic factors. The biotic factors are microorganisms that could cause diseases by affecting different parts of the crop. The most common potato diseases are early blight, late blight, and bacterial wilt. Among these crop diseases, the worst one that is challenging farmers is late blight.

In the past few years, potato disease detection has been studied by researchers in a variety of ways. The most common approach used by many studies is the application of deep learning algorithms. These algorithms have been demonstrated to be effective in detecting not only potato diseases but also various plant diseases.

As per this review work, CNN is the state-of-the-art algorithm [3, 65] which is used in numerous problems and major competitions. Unlike other traditional machine learning algorithms, CNN automatically extracts features and classifies them. Furthermore, given a sufficient amount of data, CNN learns more features resulting in better performances. Besides CNNs, there are other CV methods and algorithms used frequently in plant disease detection. In machine learning, RF, LR, k-NN, DT, LDA, SVM, and NB classifiers were used for automatic potato disease detection. Additionally, other methods like LVQ (Learning Vector Quantization), graph cut segmentation, and transfer learning via a pretrained model were used.

According to the review, deep learning algorithms have been widely employed for potato disease detection. Furthermore, CNN-based transfer learning techniques are applied to increase the performance of detection models. However, in many cases, there is no clear consensus on how the pretrained model architecture is selected and configured to build a detection model.

The review also discovered that Keras and TensorFlow are the most widely used deep learning frameworks in plant disease detection. In addition, the majority of the papers have used the open-source dataset known as the PlantVillage dataset for various plant disease detection problems. However, the dataset lacks clear data collection steps such as the stage of the leaf and other symptoms besides the leaf portion of the crop. Another gap that has not been addressed is explaining the statistical significance of the results obtained from the disease detection models. Finally, the choice of evaluation metrics in many disease detection research works appears to be overlooked.

## Figures and Tables

**Table 1:** Inclusion and exclusion criteria.

*Inclusion criteria*
1. Articles, conference papers, journals, and thesis written in English
2. Full text of #1
3. Studies that focus on CV and plant disease detection
4. Studies from the year 2016 up to 2022
5. Papers that are related to the domain of study

*Exclusion criteria*
1. Studies that are not written in English
2. Articles that do not have full access
3. Duplicated articles
4. Short papers (e.g., posters)
5. Old papers that do not satisfy the inclusion criteria set in #4

**Table 2:** Primary study selection process.

Primary study selection process	
Step 1: Selection of research repository
IEEE Xplore (IX) (270)
ScienceDirect (SD) (6,000)
Google Scholar (GS) (66,360)
Initial selection of primary studies = 72,630
Step 2: Applying exclusion criteria
Step 3: Initial selection of primary studies (80)
Step 4: Applying inclusion criteria
Step 5: Applying snowball and manual search
Step 6: Applying inclusion/exclusion criteria again
Step 7: Applying a checklist
Finally, a total of 39 primary studies were selected for data extraction and further analysis.

**Table 3:** Selected primary studies.

PS	Ref.	Title	RQ1	RQ2	RQ3
PS1	[17]	Problems and prospect of potato production and marketing—a case study on SNNPR Gamogofa zone Chencha woreda Gendogembela and Doko Yoyera rural kebele	✓		
PS2	[18]	Integrated potato (*Solanum tuberosum* L.) and late blight (*Phytophthora infestans*) disease management in Ethiopia	✓		
PS3	[19]	Recent advances in potato late blight disease management strategies	✓		
PS4	[20]	Review on potato late blight and potato tuber moth and their integrated pest management options in Ethiopia	✓		
PS5	[21]	Farmers' knowledge and practices of potato disease management in Ethiopia	✓		
PS6	[22]	Assessment of production practices of smallholder potato (*Solanum tuberosum* L.) farmers in Wolaita zone, Southern Ethiopia	✓		
PS7	[23]	Plant disease detection techniques: a review		✓	
PS8	[24]	Understanding digital image processing		✓	
PS9	[25]	Detection of plant leaf diseases using image segmentation and soft computing techniques		✓	
PS10	[26]	Detection and classification of citrus diseases in agriculture based on optimized weighted segmentation and feature selection		✓	
PS11	[27]	Detection of leaf diseases and classification using digital image processing		✓	
PS12	[28]	A survey on plant disease detection and classification using different machine learning algorithms		✓	
PS13	[29]	Plant leaf disease detection and classification based on CNN with the LVQ algorithm			✓
PS14	[30]	Using deep learning for image-based potato tuber disease detection			✓
PS15	[31]	Disease detection on the leaves of tomato plants by using deep learning			✓
PS16	[32]	Tomato plant disease classification in digital images using classification tree			✓
PS17	[33]	Deep learning for tomato diseases—classification and symptoms visualization			✓
PS18	[34]	Automatic detection and classification of leaf spot disease in sugar beet using deep learning algorithms			✓
PS19	[35]	Identification of rice diseases using deep convolutional neural networks			✓
PS20	[36]	Automated abnormal potato plant detection system using deep learning models and portable video cameras			✓
PS21	[37]	Blackleg detection in potato plants using convolutional neural networks			✓
PS22	[38]	Recognition of early blight and late blight diseases on potato leaves based on graph cut segmentation			✓
PS23	[39]	Potato leaf diseases detection using deep learning			✓
PS24	[40]	Plant identification using deep neural networks via optimization of transfer learning parameters			✓
PS25	[41]	Automatic late blight lesion recognition and severity quantification based on field imagery of diverse potato genotypes by deep learning			✓
PS26	[42]	Plant leaf detection and disease recognition using deep learning			✓
PS27	[43]	Health detection for potato leaf with convolutional neural networks			✓
PS28	[44]	Detection of potato disease using image segmentation and machine learning			✓
PS29	[45]	Disease detection of plant leaf using image processing and CNN with preventive measures			✓
PS30	[3]	Plant disease identification using transfer learning			✓
PS31	[46]	CNN-based disease detection approach on potato leaves			✓
PS32	[47]	Deep learning model for detecting and diagnosing plant disease			✓
PS33	[48]	Comparison of performance of classifiers—SVM, RF, and ANN in potato blight disease detection using leaf images			✓
PS34	[49]	Detection of potato diseases using image segmentation and multiclass support vector machine			✓
PS35	[50]	Application of transfer learning to detect potato disease from leaf image			✓
PS36	[51]	An adaptive image processing model of plant disease diagnosis and quantification based on color and texture histogram			✓
PS37	[52]	A polyhouse plant monitoring and diseases detection using CNN			✓
PS38	[53]	Potato leaf disease classification using deep learning approach			✓
PS39	[54]	Potato disease detection using machine learning			✓

**Table 4:** PS distribution before and after selection process.

Source	Count before PSS	Count after PSS
Google Scholar	66,630	9
IEEE Xplore	270	19
ScienceDirect	6,000	11
Total	**72,630**	**39**

**Table 5:** Data extraction form.

Search focus	Data item	Description
General	Identifier	Reference number given to the PSs
	Bibliography	Author, year, title, source
	Type of article	Journal article/conference paper
	Study aim	Goal of the study

**Table 6:** Data extraction form II.

RQs	Study design	Controlled experiment
RQ1	Major potato disease	Type of disease that affects potato
RQ2	Potato disease detection via CV	How CV can be used to detect potato disease
RQ3	CV algorithms/methods	CV method/algorithm for potato disease detection

**Table 7:** Quality assessment questions.

Quality assessment questions
A: Is there a clear description of the study aim?
B: Do the aim and purpose addressed through evaluation?
C: Is the target selection of documents/cases well defined?
D: Is the evidence presented enough to substantiate the claim?

**Table 8:** Summary of RQ1.

Ref.	Potato disease type	Affected crop part
[30]	BS, SS, common scab, and black dot	Potato tuber
[36]	Not mentioned	Leaf
[37]	Blackleg	Leaf
[38]	GD and SD early blight, late blight	Leaf
[39]	Early blight and late blight	Leaf
[41]	Late blight	Leaf

Note: BS = black scurf, SS = silver scurf, SD = serious degree, and GD = general degree.

**Table 9:** Summary of RQ2.

Ref.	Algorithm	Dataset	Metrics
[30]	CNN	Custom prepared	Accuracy (96%)
[36]	Fast R-CNN and YOLO v3	Custom prepared and open source	Accuracy (96.7%)
[37]	ResNet18 and ResNet50	Custom prepared	Recall (91%)
[38]	k-NN, SVM, RF, and ANN	Open source	Accuracy (97.4%)
[39]	VGG16, VGG19, Inception-v3 and LR	Open source	Accuracy (97.8%)
[41]	SegNet	Custom prepared	Not mentioned

**Table 10:** A comparison of pretrained CNN models.

Ref.	Model	*D*	Layer	*P* (M)	ER	Input size
[89]	AlexNet	8	5 convolution + 3 FC	60	16.4	227 × 227
[83]	VGG	16, 19	13–16 convolution + 3 FC	134	7.3	224 × 224
[84]	GoogLeNet	22	22 convolution, 9 inception modules	4	6.7	224 × 224
[90]	Inception-V3	48	42 convolution, 10 inception modules	22	3.5	229 × 229
[91]	ResNet	152	152 in ResNet-152	60.2	3.57	224 × 224

Note: *D* = depth, ER = error rate, FC = fully connected, *M* = millions, *P* = parameter, and all the images used are RGB.

**Table 11:** Pretrained models used in potato disease detection.

Authors	Model	# parameters	Input size
Afonso et al. [37]	ResNet18 and ResNet50	NM	224 × 224 × 3
Tiwari et al. [39]	VGG16, VGG19, and Inception-v3	NM	224 × 224 × 3
Gao et al. [41]	Encoder-decoder based on SegNet	29M	512 × 512 × 3
Lee et al. [43]	VGG16 and VGG19	138M and 143M	NM
Asif et al. [46]	AlexNet, VggNet, ResNet, and LeNet	NM	256 × 256 × 3
Sholihati et al. [53]	VGG16 and VGG19	NM	224 × 224 × 3
Sert [92]	GoogLeNet, SequezeNet, and AlexNet	NM	NM

Note: NM = not mentioned; *M* = millions.

**Table 12:** ML algorithms used in potato disease detection.

Ref.	Potato disease	Top algorithm	Result
[44]	Early blight and late blight	RF	97% accuracy
[48]	Early blight and late blight	ANN	92% accuracy
[54]	Six potato diseases	CNN	99.23% accuracy
[62]	“Not potato”	RF	98.7% accuracy

**Table 13:** A comparative analysis of studies on potato disease detection.

Author	Disease type	Algorithm	Dataset	Result
Oppenheim et al. [30]	Black scurf, silver scurf, common scab, and black dot	Custom CNN model	Custom prepared 2,465 images	96% accuracy

Oishi et al. [36]	Not mentioned	Fast R-CNN and YOLO v3	Pascal VOC 2007, COCO dataset, PlantVillage, and custom prepared	Fast R-CNN with 96.7% accuracy

Afonso et al. [37]	Blackleg	ResNet18 and ResNet50	Custom prepared (532 images)	91% recall

Hou et al. [38]	General degree and a serious degree of both early blight and late blight	k-NN, SVM, RF, and ANN	AI challenger global AI contest (2840 images)	SVM with 97.4% accuracy

Tiwari and Divyansh [39]	Early blight and late blight	VGG16, VGG19, Inception-v3, and LR	PlantVillage dataset (2,152)	VGG19 with LR 97.8% accuracy, 97.8% precision, 97.8% recall, and 97.8% F1-score

Gao et al. [41]	Late blight	SegNet	Custom prepared 2,100 images	Not mentioned

Lee et al. [43]	Early blight	Proposed model using CNN, VGG16, and VGG19	Not mentioned	The proposed model scored 99% accuracy

Iqbal and Talukder [44]	Early blight and late blight	RF, LR, k-NN, DT, NB, LDA, and SVM	Custom prepared 450 images	RF scored 97% accuracy

Asif et al. [46]	Early blight and late blight	AlexNet, VggNet, ResNet, LeNet, and sequential model	Kaggle, dataquest dataset, and custom prepared dataset	The proposed CNN model scored 97% accuracy

Patil et al. [48]	Early blight and late blight	SVM, RF, and ANN	Custom prepared 892 images and PlantVillage (300 images)	ANN scored 92% accuracy
Sholihati et al. [53]	Alternaria solani, phytophthora infestans, virus, and insect	VGG16, proposed model, and VGG19	5,200 open-source datasets	Proposed model 91% accuracy, 88% precision, and 89% recall

Tarik et al. [54]	Roll virus, hollow heart, scab, soft rot, sutali poka rrog, virus jonito rog, and early blight	Custom-built CNN	Custom prepared 2034 images	99.23% accuracy

Sert [92]	Early blight and late light	Faster R-CNN and GoogLeNet, SequezeNet, and AlexNet	Plant village and custom prepared dataset	Faster R-CNN with GoogLeNet scored 98.06% accuracy, 98% precision, 98% recall, and 98% F1-score

Rashid et al. [97]	Early blight and late blight	Custom-built CNN	Custom prepared 4062 images	99.75% accuracy, 99.6% precision, 99.6% recall, and 99.6% F1-score

**Table 14:** Terms frequency.

Word	Count	Relevance
Deep learning	10	0.99
Convolutional neural network	3	0.74
Potato disease	5	0.49
Detection of plant	2	0.49
Detection of potato	2	0.49
Plant disease detection	2	0.49
Deep learning model	2	0.49

## Data Availability

No underlying data was collected or produced in this study.
